# Mouth rinsing and ingesting salty or bitter solutions does not influence corticomotor excitability or neuromuscular function

**DOI:** 10.1007/s00421-023-05141-3

**Published:** 2023-01-26

**Authors:** Edward Gray, Rocco Cavaleri, Jason Siegler

**Affiliations:** 1 School of Health Sciences, Western Sydney University, Campbelltown, Australia; 2 Brain Stimulation and Rehabilitation (BrainStAR) Lab, School of Health Sciences, Western Sydney University, Campbelltown, Australia; 3 College of Health Solutions, Arizona State University, Tempe, USA

**Keywords:** Taste, Unpleasant, Quinine, Exercise, Ergogenic aid

## Abstract

**Purpose:**

To explore the effect of tasting unpleasant salty or bitter solutions on lower limb corticomotor excitability and neuromuscular function.

**Methods:**

Nine females and eleven males participated (age: 27 ± 7 years, BMI: 25.3 ± 4.0 kg m^−2^). Unpleasant salty (1 M) and bitter (2 mM quinine) solutions were compared to water, sweetened water, and no solution, which functioned as control conditions. In a non-blinded randomized cross-over order, each solution was mouth rinsed (10 s) and ingested before perceptual responses, instantaneous heart rate (a marker of autonomic nervous system activation), quadricep corticomotor excitability (motor-evoked potential amplitude) and neuromuscular function during a maximal voluntary contraction (maximum voluntary force, resting twitch force, voluntary activation, 0–50 ms impulse, 0–100 impulse, 100–200 ms impulse) were measured.

**Results:**

Hedonic value (water: 47 ± 8%, sweet: 23 ± 17%, salt: 71 ± 8%, bitter: 80 ± 10%), taste intensity, unpleasantness and increases in heart rate (no solution: 14 ± 5 bpm, water: 18 ± 5 bpm, sweet: 20 ± 5 bpm, salt: 24 ± 7 bpm, bitter: 23 ± 6 bpm) were significantly higher in the salty and bitter conditions compared to control conditions. Nausea was low in all conditions (< 15%) but was significantly higher in salty and bitter conditions compared to water (water: 3 ± 5%, sweet: 6 ± 13%, salt: 7 ± 9%, bitter: 14 ± 16%). There was no significant difference between conditions in neuromuscular function or corticomotor excitability variables.

**Conclusion:**

At rest, unpleasant tastes appear to have no influence on quadricep corticomotor excitability or neuromuscular function. These data question the mechanisms via which unpleasant tastes are proposed to influence exercise performance.

## Introduction

It is well established that an athlete's nutrition influences their recovery and performance during training and competition (Thomas et al. 2016). Logically, and in general, nutritional substances must first be ingested and digested before its benefits are apparent. However, emerging evidence suggests that tasting certain substances, without ingestion, can influence exercise performance (for review, see: Best et al. 2021). This was first demonstrated by Carter et al. (2004) who showed that mouth rinsing a carbohydrate solution, and then expectorating, improved 1 h cycling time trial performance by 2.9%. However, the mechanisms via which carbohydrate mouth rinsing promotes ergogenic effects remain equivocal. It is proposed that carbohydrate is detected by unidentified oral receptors (Chambers et al. 2009) whose afferents increase activation in brain regions involved with motivation and motor control (Chambers et al. 2009; Turner et al. 2014). Subsequently, this may increase corticomotor excitability (Gant et al. 2010) and neuromuscular function (Jeffers et al. 2015).

The ergogenic effect of carbohydrate mouth rinsing highlights the possibility that other tastes could also influence exercise performance. Indeed, tasting caffeine, and also menthol, has been shown to have a positive effect on exercise performance (Ehlert et al. 2020; Gavel et al. 2021). In addition to these compounds, mouth rinsing and ingesting quinine, a strong bitter taste, has been shown to improve mean 30 s cycling sprint power by 2.4% and 3.9% compared to sweet and water solutions, respectively (Gam et al. 2014). Despite the large performance improvement, the mechanisms that facilitate performance changes after tasting quinine are unclear. A proposed mechanism is that quinine could increase corticomotor excitability, and therefore enhance motor output. Supporting this, Gam et al. (2015a) found mouth rinsing and ingesting quinine to increase corticomotor excitability by 16% in the resting first dorsal interossei (FDI) muscle. However, it is unclear if the large change in corticomotor excitability would be similar in contracting lower limb muscles that are relevant to exercise performance. Indeed, the neural pathways that influence corticospinal excitability are task specific (Kalmar 2018) and intracortical and corticospinal control are different between upper and lower limb muscles (Chen et al. 1998). For example, noxious stimuli (such as pain) are thought to inhibit upper limb motor-evoked potentials while facilitating lower limb excitability (Rice et al. 2021), highlighting limb-specific corticomotor responses to negative or unpleasant stimuli.

It is unknown if quinine’s ergogenic property is attributable to the compound itself, its bitterness, or its unpleasantness. Tasting quinine evokes a significant autonomic nervous system (ANS) response (Rousmans 2000) that may support its effect on exercise performance by increasing heart and breathing rate, redistributing blood flow and by directly influencing muscle fibre contractility (Roatta and Farina 2010). Interestingly, the ANS response to quinine ingestion may be mediated by its hedonic value and, therefore, also occurs after tasting other unpleasant tastes (Rousmans 2000). Therefore, it is plausible that unpleasant tastes beyond quinine could also have ergogenic potential. Indeed, it has recently been shown that an unpleasant salt mouth rinse can ameliorate neuromuscular fatigue after cycling exercise (Khong et al. 2020).

The mechanisms by which tasting unpleasant tastes could be ergogenic are not well understood. It is unclear if increased corticomotor excitability in the resting FDI after tasting quinine (Gam et al. 2015a) is translatable to contracting lower limb muscles. Furthermore, it is unknown if changes in corticomotor excitability would be sufficient to influence lower limb neuromuscular function that would be relevant to exercise performance. Additionally, it is unclear if tastes other than quinine, which are similarly unpleasant, could also influence these mechanistic pathways. Therefore, the purpose of the present study was to determine the influence of tasting unpleasant bitter and salty solutions on quadricep corticomotor excitability and neuromuscular function. We hypothesized that, compared to control conditions, the unpleasant tastes would increase corticomotor excitability, facilitating enhanced neuromuscular function.

## Methods

### Study design

Eleven male (mean ± SD, age: 30 ± 8 years, BMI: 24.3 ± 3.3 kg m^−2^) and nine female (age: 25 ± 4 years, BMI: 26.4 ± 4.7 kg m^−2^) healthy individuals volunteered to participate and provided written informed consent. Inclusion criteria were: aged 18 to 50 years, non-smoker and did not have any gustatory or olfactory disorders. Experimental procedures were conducted in accordance with the Declaration of Helsinki after approval by Western Sydney University Human Research Ethics Committee (approval number H13932).

The present study used a non-blinded randomized cross-over design to assess the influence of unpleasant tastes on corticomotor excitability and neuromuscular function. No solution, water, and sweet conditions were controls for the unpleasant salt and bitter solutions. Participants visited the laboratory once to complete a three-phase experiment. First, participants' perceptual and heart rate (a marker of ANS activation) responses to the tastes were determined. Second, the excitability of the quadriceps corticomotor representation was assessed using single pulse transcranial magnetic stimulation (TMS). Third, neuromuscular function was evaluated using knee extensor maximal voluntary contractions (MVCs) with peripheral nerve stimulation.

### Experimental trial

Participants abstained from exercise on the day of the trial and from food or drink (except water) in the hour before the trial began. To increase the accuracy of each measurement, participants were presented each of the five conditions (see Intervention Conditions) twice per phase and the average of both presentations was calculated. Participants received the first presentation of each condition in a randomized order and then the second presentation of each condition in a randomized order. In all phases, measurements were taken immediately after each solution was mouth rinsed (10 s) and ingested with 5 min between each presentation. A 10 s mouth rinse was chosen to replicate prior research investigating the physiological and performance effects of tasting quinine (Gam et al. 2014; Gam et al. 2015a, b). Five minutes between conditions was chosen, because the physiological response to the conditions was expected to be transient (~ 1 min; Gam et al. 2014; Gam et al. 2015a, b; Rousmans 2000) and this would allow sufficient washout between solutions. Participants drank room temperature water ad libitum between presentations. Furthermore, once all measurements were completed after each quinine ingestion, participants ingested a low concentration salt solution (0.1 M, 25 ml) to neutralize the long-lasting bitter taste before the next solution was ingested (Gam et al. 2014).

### Intervention conditions

In the present study, because the independent variable was taste, it was impossible to blind participants to condition and remove the possibility of a placebo or nocebo effect. To mitigate this, we included a sweet and a water condition which we expected individuals to perceive as ergogenic and neutral, respectively. Then, before the experimental trial, participants completed a questionnaire determining current perceptions regarding how the different tastes would influence neuromuscular function. While not removing the influence of any placebo or nocebo effect on the unpleasant tastes, these steps enabled the extent of a placebo or nocebo effect to be measured and compared to control conditions.

During the experiment, all solutions were mouth rinsed before being ingested. Ingestion was undertaken, because many of the bitter-specific taste receptors are located on the posterior tongue and upper gastrointestinal tract (Behrens et al. 2007) and, therefore, may only be activated once the solution is ingested. Supporting this, mouth rinsing and ingesting a bitter solution has been shown to improve cycling sprint performance (Gam et al. 2014), whereas mouth rinsing alone does not have an effect (Gam et al. 2015b). Therefore, in the present study, to ensure activation of bitter-specific taste receptors and to control for the method of solution delivery, all solutions were ingested after being mouth rinsed (10 s).

All conditions were presented in red 50 ml plastic containers at room temperature. Solutions were served as 25 ml except the ‘no solution’ condition which was a 25 g mass. In the no solution condition, the container was raised to the mouth to replicate the movement that occurred in the other conditions. The sweet condition was 14% artificial sweetener (Cottees Apple and Raspberry Cordial, Schweppes; Melbourne, Australia). The bitter condition was a 2 mM quinine hydrochloride dihydrate solution (Sigma-Aldrich; Missouri, USA) to match the concentration used in previous research (Gam et al. 2014, 2015a). As no research has previously sought to determine an appropriate concentration for salt tasting, we conducted a pilot test with six individuals to determine the highest concentration of salt solution (between 0.25 M and 2 M) that did not induce significant nausea when mouth rinsed (10 s) and ingested. Based on these results (data not shown), we chose to use a 1 M salt solution (Table Salt; Coles, Melbourne, Australia) in the present study.

### Phase 1: physiological and perceptual responses

Physiological and perceptual responses to each condition were measured together. To minimize distractions, participants were seated at a desk facing a blank wall with noise cancelling headphones (Bose QuietComfort; Massachusetts, USA) playing brown noise. The procedure began with 15 min of rest during which participants became accustomed to their surroundings. Then, a remote-activated yellow light signalled to participants to slowly raise the solution and rinse it around the mouth (10 s) until the light was turned off. Participants were instructed to remain as still as possible for 1 min, while physiological measures were taken. Throughout the procedure, instantaneous heart rate was measured beat by beat using finger photoplethysmography (Finometer Pro; Finapres Medical Systems, Enschede, The Netherlands). Change in (∆) heart rate was then calculated as the difference between the maximum value after mouth rinsing the solution and the average 10 s value immediately preceding the mouth rinse.

Once physiological parameters returned to baseline (1 min after solution ingestion), participants then completed perceptual questionnaires. Hedonic value and nausea were assessed using 10 cm visual analogue scales anchored by the labels 'highly pleasant’ and ‘highly unpleasant’, or ‘no nausea’ and ‘extreme nausea’, respectively. Unpleasantness and taste intensity were assessed using 20 cm adjusted general labelled magnitude scales (Green et al. 1996).

The influence of taste on physiological markers of ANS activation (e.g., heart rate) has previously been documented (Gam et al. 2014; Rousmans 2000). However, this has not been assessed using the exact concentrations and quantities used in the present study. Therefore, instantaneous heart rate was assessed alongside perceptual measures to confirm that the bitter and salt solutions induced a similar ANS disturbance.

### Phase 2: transcranial magnetic stimulation

Prior to participating, all participants were screened using the adult TMS safety screening questionnaire (Rossi et al. 2009, 2011). During the TMS phase, participants were seated with their head and arms supported and the lower shank restrained. Electromyography (EMG) activity was recorded using adjacent unipolar Ag/AgCl EMG electrodes (MLA1010; ADinstruments, Sydney, Australia) located on the muscle belly of the right vastus lateralis (VL) and vastus medialis (VM). The VL and VM inferior electrodes were placed 8–12 cm and 3–5 cm above the patella, respectively (Hermens et al. 2000). A ground electrode was placed on the tibial tuberosity. Electrode placement was confirmed via palpation by a qualified physiotherapist. Electrode sites were shaved, abraded, and cleaned with an alcohol swab prior to placement. EMG signals were amplified, band pass filtered (20–1000 Hz), and sampled at 2000 Hz using a Power 1401 Data Acquisition System and then processed in Signal5 (Cambridge Electronic Design; Cambridge, UK).

Maximal VL EMG activity was determined during a knee extensor MVC. Subsequently, all TMS pulses were delivered during voluntary contractions at 10% of maximal VL EMG activity. To ensure consistency, participants were provided real-time visual feedback of VL EMG activity. Single-pulse TMS was delivered using a Magstim Super Rapid^2^ Plus^1^ (Whitland, UK) with an air-cooled figure of eight coil (Cavaleri et al. 2018; van de Ruit et al. 2015). The coil was placed over the left cranial hemisphere, tangential to the scalp, with the handle positioned posterior-laterally at a ~ 45 angle to the midline to induce posterior-lateral to anterior-medial second-phase current (Richter et al. 2013). The optimal site for stimulation (hotspot) was determined as the position of the coil that facilitated the largest motor-evoked potentials (MEPs) in the VL (Groppa et al. 2012; Rossini et al. 2015). The hotspot coil position was recorded using Brainsight software (Rogue Research Inc, Quebec, Canada) to ensure replication of stimulation site between conditions. The active motor threshold was then determined as the lowest coil stimulation intensity that elicited MEPs clearly discernible from background EMG activity in both the VL and VM (Groppa et al. 2012; Rossini et al. 2015). MEPs in both the VM and VL were recorded following stimulation of the combined hotspot. Stimulation intensity was 110% of active motor threshold for all experimental trials (Cavaleri et al. 2017), corresponding to a stimulus intensity of 86 ± 14% of maximum stimulator output.

For each experimental condition, the solution was mouth rinsed (10 s) and then ingested, followed immediately by 10 TMS pulses over the hotspot with a 3 s interstimulus interval (Groppa et al. 2012). Although recent evidence recommends a greater number of stimuli during single site TMS investigations (Brownstein et al. 2018), we delivered 10 stimuli as we expected the physiological response to solution ingestion to be transient (Gam et al. 2014, 2015a, b; Rousmans 2000). Thus, longer protocols with more stimuli were avoided to ensure that corticomotor excitability did not vary during MEP acquisition itself. Notably though, because each condition was measured twice, the data presented for each condition are the average of 20 MEPs in total. Furthermore, it has been shown that ten MEPs are sufficient to achieve excellent within-session reliability in the lower limb (Cavaleri et al. 2017; Lewis et al. 2014). We also assessed the reliability of the TMS procedure in our laboratory by taking three baseline measurements prior to introducing the experimental conditions. These measurements demonstrated that MEP recordings from both VL and VM had good reliability (ICC = 0.99, coefficient of variation [CV] ≤ 10%), comparable to previous reliability research (Temesi et al. 2017), but better than others (Brownstein et al. 2018; Leung et al. 2018). MEP amplitude was calculated as the RMS (root-mean-square) EMG amplitude between the visually identified MEP onset and offset following each TMS pulse. Background EMG from 55 to five milliseconds prior to stimulation was subtracted. The mean of the ten MEP recordings was used during data analysis.

### Phase 3: neuromuscular function

Neuromuscular function was assessed using knee extensor MVC’s with peripheral nerve stimulation on a custom-built dynamometer chair. Participants sat on the chair with ninety-degree hip and knee angles. A thinly padded cuff, connected to a force transducer, was tightly secured above the lateral malleolus. Force was sampled at 4000 Hz (Powerlab; AD Instruments, Sydney, Australia). EMG electrodes from the TMS procedure remained on the VM and VL for the neuromuscular function testing. EMG signals were sampled at 4000 Hz (Powerlab; AD Instruments, Sydney, Australia) and band pass filtered (20–500 Hz).

For femoral nerve stimulation, anode and cathode gel electrodes (Red Dot; 3 M, Minnesota, USA) were placed on the gluteus maximus at the midpoint between the iliac crest and the femoral head, and within the femoral triangle, respectively. The optimal position of the cathode was determined by stimulating at 30 mA using a portable cathode probe in various positions within the femoral triangle. The cathode electrode was then placed in the position that elicited the greatest knee extensor contraction. Maximum stimulation intensity was then determined by incrementally (20 mA) increasing the current until the resultant resting twitch force plateaued. Test stimulation intensity during the experimental trial was set at 130% of the maximum intensity, corresponding to a current of 187 ± 78 mA. Electrical stimulations were initiated during, and 2–3 s after each MVC, as 0.2 ms square waves applied as 10 Hz doublets. We chose 10 Hz doublets to reduce participant discomfort and to allow clear separation of the M-waves. 10 Hz doublets allow summation of the quadricep twitch force and previous research has found no difference in potentiated twitch force or voluntary activation trends between 10 and 100 Hz doublet stimulation (Marshall et al. 2015; Metcalf et al. 2019).

After a self-selected warm-up, participants were familiarized with the procedure by performing 2–3 MVCs without stimulation and then 2–3 MVCs with stimulation. Each MVC was 2–3 s long, and participants were instructed to contract as hard and as fast as possible. For each experimental condition, the solution was mouth rinsed (10 s) and ingested followed immediately by an MVC. To determine the reliability of the procedure in our laboratory, three baseline measurements were taken prior to introducing the experimental conditions. Most neuromuscular function variables had a CV of ≤ 10% which is comparable to previous reliability work (Place et al. 2007; Todd et al. 2004). However, all impulse variables were less reliable (CV > 10%), as is common with impulse measurements (Courel-Ibáñez et al. 2020).

Measures of maximum voluntary force, potentiated twitch force, voluntary activation, and impulse were taken for each MVC. Maximum voluntary force was defined as the highest force throughout the MVC excluding the interpolated twitch. Voluntary activation (Merton 1954) was estimated using (Strojnik and Komi 1998): $100-\left(D\times \left(\frac{\mathrm{IT}}{\mathrm{MVF }\times \mathrm{PT}}\right)\right)\times 100$ where *D* is the difference between the force just before the interpolated twitch (IT) and the maximum force during the interpolated twitch, MVF is maximum voluntary force, and PT is the maximum force during the potentiated twitch. Impulse was calculated using the trapezium rule for 0–50 ms, 0–100 ms, and 100–200 ms from contraction onset. Contraction onset was defined when the baseline force was exceeded by 5 N.

For each MVC, the EMG signal was processed to calculate EMG Max and peak to peak M-wave amplitude for the VM and VL. EMG Max was calculated as the highest 0.25 s averaged root-mean-square EMG between contraction onset and the interpolated twitch.

### Data analysis

Three participants did not pass the TMS screen, and for two participants, clear MEPs could not be determined at 100% of maximal stimulator output. Therefore, these individuals did not undertake the TMS phase of the experiment and MEP data are only presented for 15 participants. Three participants could not withstand the discomfort of the peripheral nerve stimulation and completed the neuromuscular function assessment but without electrical stimulation. Therefore, voluntary activation, potentiated twitch force, and neuromuscular function EMG data contain only 17 datasets. All other variables include data from all 20 participants. A sensitivity analysis was performed post hoc, showing that we could detect medium effect sizes; ES > 0.68, ES > 0.63, ES > 0.58 with 80% statistical power, and *α* = 0.05 for a sample of *n* = 15, *n* = 17 and *n* = 20, respectively. Between individual comparisons (male versus female) were not analysed in the present study as this was not the primary aim and our sample was not appropriately powered to conduct these analyses.

Statistical analysis was conducted using SPSS (IBM; Armonk, New York). To increase the accuracy of each measurement, the mean of the first and second presentation of each condition was analysed. Sphericity was assessed using Mauchly’s Test of Sphericity. Significant differences between conditions were assessed using a one-way repeated-measures ANOVA, with Greenhouse–Geisser adjustment where appropriate. Following a significant outcome, Bonferroni post hoc tests determined which conditions were significantly different. Data within the text are presented as mean difference ± 95% confidence interval (mean ∆ ± 95% CI) alongside Cohens d effect sizes (ES).

In addition to differences between conditions, differences between the first and second presentation of each condition were assessed using paired t tests to determine the existence of order effects. For variables with significant order effects, the first presentation of each condition and the second presentation of each condition were analysed separately using a one-way repeated-measures ANOVA and the post hoc tests previously described. For all analysis, *α* = 0.05.

## Results

### Perceptual

The pre-trial questionnaire showed that 10/20 participants were unsure how the tastes would influence neuromuscular function and 3/20 thought the tastes would have no influence. Of the 7/20 participants who thought one or more of the tastes would influence neuromuscular function, 6/20 thought the sweet, 3/20 the salt and 1/20 thought the bitter solution would improve neuromuscular function. 4/20 individuals thought the bitter, 1/20 water and 1/20 thought the salt would impair neuromuscular function.

All data are shown in Table 1. There were no order effects in any of the measured perceptual variables (*p* > 0.05). There was a significant effect of condition on hedonic value (*F* (3, 57) = 86.7, *p* < 0.001, *η*^2^ = 0.82), taste intensity (*F* (3, 57) = 73.3, *p* < 0.001, *η*^2^ = 0.80), unpleasantness (*F* (2.22, 42.11) = 77.6, *p* < 0.001 *η*^2^ = 0.80), and nausea (*F* (2.22, 41.41) = 7.2, *p* = 0.002, *η*^2^ = 0.28). Hedonic value was significantly higher in salt and bitter conditions compared to water (salt: *p* < 0.001, mean ∆ ± 95% CI 24 ± 8%, ES = 2.91; bitter: *p* < 0.001, mean ∆ ± 95% CI 33 ± 9%, ES = 3.66) and sweet (salt: *p* < 0.001, mean ∆ ± 95% CI 48 ± 14%, ES = 3.60; bitter: *p* < 0.001, mean ∆ ± 95% CI 57 ± 16%, ES = 4.12). There was no significant difference in hedonic value between the unpleasant salty and bitter tastes (*p* = 0.074, mean ∆ ± 95% CI 9 ± 10%, ES = 0.99). Similarly, taste intensity was significantly higher in salt and bitter conditions compared to sweet (salt: *p* = 0.004, mean ∆ ± 95% CI 18 ± 13%, ES = 1.31; bitter: *p* < 0.001, mean ∆ ± 95% CI 31 ± 12%, ES = 1.84) and water (salt: *p* < 0.001, mean ∆ ± 95% CI 42 ± 10%, ES = 4.20; bitter: *p* < 0.001, mean ∆ ± 95% CI 55 ± 13%, ES = 3.97). Furthermore, ratings of unpleasantness were significantly higher in salt and bitter conditions compared to sweet (salt: *p* < 0.001, mean ∆ ± 95% CI 35 ± 13%, ES = 2.64; bitter: *p* < 0.001, mean ∆ ± 95% CI 53 ± 15%, ES = 3.25) and water (salt: *p* < 0.001, mean ∆ ± 95% CI 39 ± 11%, ES = 3.39; bitter: *p* < 0.001, mean ∆ ± 95% CI 56 ± 14%, ES = 3.84). However, taste intensity and unpleasantness were significantly higher in bitter compared to salt (*p* = 0.046, mean ∆ ± 95% CI 13 ± 13%, ES = 0.76 and *p* = 0.033, mean ∆ ± 95% CI 17 ± 17%, ES = 0.94, respectively). Nausea was low in all conditions (< 15/100) but was significantly higher in salt and bitter conditions compared to water (salt: *p* = 0.024, mean ∆ ± 95% CI 5 ± 5%, ES = 0.63; bitter: *p* = 0.013, mean ∆ ± 95% CI 11 ± 9%, ES = 0.95).

**Table 1:** Perceptual responses following mouth rinsing and ingesting water, sweet, salt, and bitter solutions

	Water	Sweet	Salt	Bitter
Hedonic value	47 ± 8^b,c,d^	23 ± 17^a,c,d^	71 ± 8^a,b^	80 ± 10^a,b^
Taste intensity	2 ± 3^b,c,d^	26 ± 14^a,c,d^	44 ± 14^a,b,d^	57 ± 19^a,b,c^
Unpleasantness	2 ± 3^c,d^	5 ± 10^c,d^	41 ± 16^a,b,d^	58 ± 21^a,b,c^
Nausea	3 ± 5^c,d^	6 ± 13	7 ± 9^a^	14 ± 16^a^

Data presented as mean ± SD (*n* = 20)

^a, b, c, d^ Significantly different to water, sweet, salt, and bitter, respectively

### Physiological

**Fig. 1:**
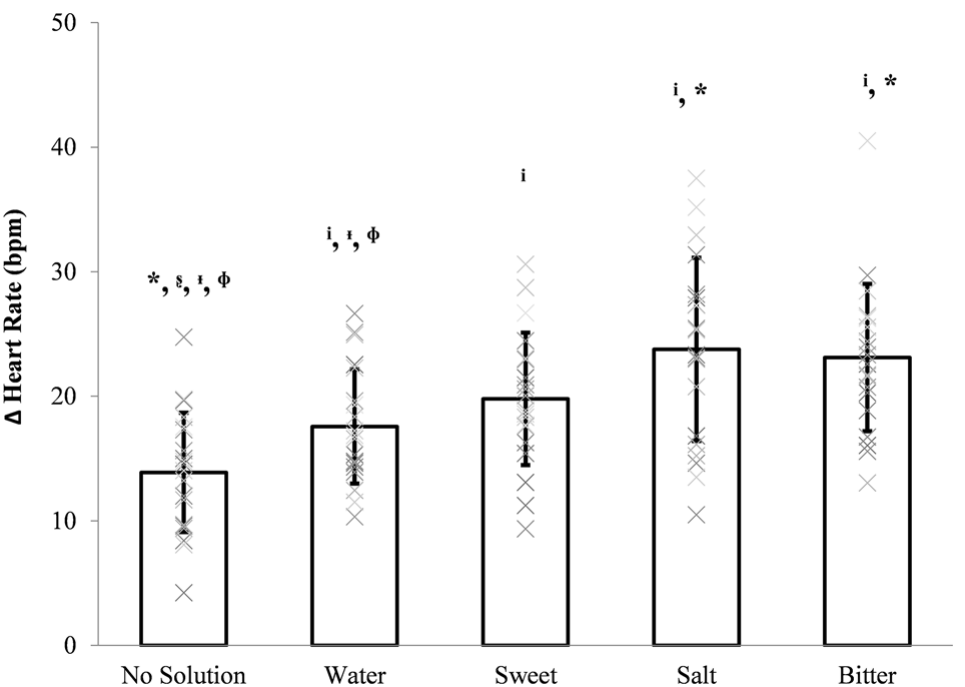
∆ heart rate immediately following mouth rinsing and ingesting water, sweet, salt, bitter, or no solution control. Data presented as mean ± SD (*n* = 20). Crosses represent individual responses. i, *, , ,  denote significantly different to no solution, water, sweet, salt, and bitter, respectively

There was no order effect for changes in heart rate (*p* > 0.05). Heart rate increased in all conditions. There was a significant effect of condition on ∆ heart rate (Fig. 1; *F* (4, 76) = 16, *p* < 0.001, *η*^2^ = 0.46). Post hoc tests showed that the increase in heart rate was significantly greater in salt and bitter conditions compared to no solution (salt: *p* < 0.001, mean ∆ ± 95% CI 10 ± 5 bpm, ES = 1.59; bitter: *p* < 0.001, mean ∆ ± 95% CI 9 ± 5 bpm, ES = 1.71) and water (salt: *p* = 0.018, mean ∆ ± 95% CI 6 ± 6 bpm, ES = 1.01; bitter: *p* = 0.012, mean ∆ ± 95% CI 5 ± 5 bpm, ES = 1.05). The increase in heart rate was also significantly higher in sweet compared to no solution (*p* = 0.004, mean ∆ ± 95% CI 6 ± 5 bpm, ES = 1.17). There was no significant difference in ∆ heart rate between sweet and salt (*p* = 0.19, mean ∆ ± 95% CI 4 ± 5 bpm, ES = 0.62), sweet and bitter (*p* = 0.24, mean ∆ ± 95% CI 3 ± 5 bpm, ES = 0.59), and bitter and salt (*p* = 1.00, mean ∆ ± 95% CI 1 ± 4 bpm, ES = 0.10) conditions.

### Corticomotor excitability

There was no order effect for VM or VL MEP RMS amplitude (*p* > 0.05). There was no significant difference between conditions in VL or VM MEP RMS amplitude (Table 2; *F* (1.88, 26.36) = 0.41, *p* = 0.657, *η*^2^ = 0.03; *F* (4, 56) = 0.68, *p* = 0.608, *η*^2^ = 0.05).

**Table 2:** VL and VM MEP’s during submaximal knee extensor contraction immediately following mouth rinsing and ingesting water, sweet, salt, bitter, or no solution control

	CV	Control	Water	Sweet	Salt	Bitter
VL MEP (mV)	8%	0.18 ± 0.19	0.20 ± 0.22	0.19 ± 0.20	0.19 ± 0.20	0.19 ± 0.22
VM MEP (mV)	10%	0.14 ± 0.11	0.14 ± 0.13	0.14 ± 0.11	0.15 ± 0.13	0.13 ± 0.10

Data presented as mean ± SD (*n* = 15)

### Neuromuscular function

There was no significant effect of order on voluntary activation, VL M-wave and VM M-wave amplitude (*p* > 0.05). However, the second presentation of each condition was significantly lower than the first presentation in maximum voluntary force (average change − 6%), resting twitch force (average change − 5%), 0–50 ms impulse (average change − 6%), 0–100 ms impulse (average change − 8%), 100–200 impulse (average change − 6%), VL EMG Max (average change − 7%), and VM EMG Max (average change − 6%). In addition to analysing the average of both presentations, for all variables with an order effect, the first and second presentations were analysed separately.

For the first presentation of each condition, there was no significant difference in maximum voluntary force (*F* (4, 64) = 1.530, *p* = 0.204, *η*^2^ = 0.09), resting twitch force (*F* (4, 64) = 1.188, *p* = 0.324, *η*^2^ = 0.10), 0–50 ms impulse (*F* (4, 64) = 1.753, *p* = 0.149, *η*^2^ = 0.10), 0–100 ms impulse (*F* (4, 64) = 1.774, *p* = 0.145, *η*^2^ = 0.10), 100–200 ms impulse (*F* (2.49, 39.83) = 2.108, *p* = 0.124, *η*^2^ = 0.12), VL EMG Max (*F* (4, 64) = 1.693, *p* = 0.163, *η*^2^ = 0.10), and VM EMG Max (*F* (4, 64) = 2.188, *p* = 0.08, *η*^2^ = 0.12). For the second presentation of each condition, there was no significant difference in maximum voluntary force (*F* (4, 64) = 1.699, *p* = 0.161, *η*^2^ = 0.10), resting twitch force (*F* (2.51, 40.11) = 1.516, *p* = 0.229, *η*^2^ = 0.09), 0–50 ms impulse (*F* (2.37, 37.89) = 0.909, *p* = 0.426, *η*^2^ = 0.0.05), 0–100 ms impulse (*F* (2.53, 40.55) = 0.734, *p* = 0.516, *η*^2^ = 0.04), 100–200 ms impulse (*F* (2.49, 39.83) = 0.618, *p* = 0.651, *η*^2^ = 0.04), VL EMG Max (*F* (4, 64) = 0.707, *p* = 0.590, *η*^2^ = 0.04), and VM EMG Max (*F* (4, 64) = 0.759, *p* = 0.556, *η*^2^ = 0.05).

For the average of both presentations, there was no significant difference in maximum voluntary force (*F* (4, 76) = 1.008, *p* = 0.409, *η*^2^ = 0.05), resting twitch force (*F* (4, 60) = 0.593, *p* = 0.669, *η*^2^ = 0.04), voluntary activation (*F* (4, 60) = 16, *p* = 0.340, *η*^2^ = 0.07), 0–50 ms impulse (*F* (4, 76) = 1.089, *p* = 0.368, *η*^2^ = 0.05), 0–100 ms impulse (*F* (4, 76) = 1.309, *p* = 0.274, *η*^2^ = 0.06), or 100–200 ms impulse (*F* (4, 76) = 1.650, *p* = 0.170, *η*^2^ = 0.08) between conditions (Table 3). Furthermore, there was no significant difference in VL or VM M-wave (*F* (2.25, 33.70) = 0.969, *p* = 0.399, *η*^2^ = 0.06; *F* (1.88, 28.17) = 0.352, *p* = 0.693, *η*^2^ = 0.02; respectively) or EMG Max (*F* (4, 60) = 2.088, *p* = 0.094, *η*^2^ = 0.12; *F* (4, 60) = 0.663, *p* = 0.620, *η*^2^ = 0.04; respectively) between conditions (Table 4).

**Table 3:** Neuromuscular function data during knee extensor MVCs immediately following mouth rinsing and ingesting water, sweet, salt, bitter, or no solution control

	CV (%)	Control	Water	Sweet	Salt	Bitter
Maximum voluntary force (*N*)	6	469 ± 132	471 ± 138	475 ± 145	461 ± 142	470 ± 140
Resting twitch force (*N*)	3	205 ± 50	203 ± 48	201 ± 46	202 ± 48	204 ± 49
Voluntary activation (%)	5	91 ± 10	93 ± 8	92 ± 8	91 ± 8	92 ± 7
0–50 ms impulse (Ns)	21	3.3 ± 1.2	3.5 ± 1.3	3.7 ± 1.0	3.5 ± 1.3	3.4 ± 1.0
0–100 ms impulse (Ns)	18	12.8 ± 5.0	13.8 ± 5.1	14.1 ± 4.8	13.2 ± 5.0	13.6 ± 4.9
100–200 ms impulse (Ns)	11	31.0 ± 10.4	32.4 ± 10.7	32.6 ± 10.3	31.1 ± 10.2	32.1 ± 10.5

Data presented as mean ± SD (*n* = 17)

**Table 4:** VL and VM EMG data during knee extensor MVC immediately following mouth rinsing and ingesting water, sweet, salt, bitter, or no solution control

	CV (%)	Control	Water	Sweet	Salt	Bitter
VL M-wave amplitude (mV)	7	8.7 ± 5.2	8.9 ± 5.3	8.9 ± 5.3	8.9 ± 5.2	8.9 ± 5.4
VM M-wave amplitude (mV)	6	8.5 ± 4.7	8.6 ± 4.7	8.5 ± 4.7	8.4 ± 4.4	8.5 ± 4.5
VL EMG max (mV)	10	0.58 ± 0.29	0.60 ± 0.33	0.62 ± 0.33	0.59 ± 0.32	0.59 ± 0.30
VM EMG max (mV)	9	0.54 ± 0.27	0.55 ± 0.27	0.55 ± 0.29	0.52 ± 0.24	0.54 ± 0.28

Data presented as mean ± SD (*n* = 17)

## Discussion

Prior research has found tasting an unpleasant bitter quinine solution to exhibit ergogenic potential by enhancing cycling sprint performance (Gam et al. 2014). In the present study, we explored some of the purported mechanisms that may explain the observed ergogenic effect of tasting quinine. Specifically, we investigated if quinine ingestion could increase knee extensor corticomotor excitability and enhance neuromuscular function. Additionally, we investigated if a similar unpleasant salt solution influenced these same mechanistic pathways. The salt and bitter solutions had similar perceptual responses and induced similar changes in heart rate, indicating a similar ANS disturbance. However, we found mouth rinsing and ingesting neither quinine nor salt to influence quadricep neuromuscular function or corticomotor excitability. These data do not have direct practical application to exercise performance; however, these data question the supposed mechanisms via which quinine, and other unpleasant tastes, may benefit exercise performance.

In the present study, there was no significant difference in VM or VL motor-evoked potentials, during a 10% of MVC contraction, between the unpleasant bitter or salt solutions and the control conditions. Conversely, Gam et al. (2015a) found mouth rinsing and ingesting quinine and water to increase first dorsal interosseous mean motor-evoked potentials by 16% and 10%, respectively, when the muscle was at rest. The apparent discrepancy could be explained by differences in the muscle group measured and the activity of the muscle. Upper limb muscles are, in general, involved with fine control of small muscle movements, whereas lower limb muscles are involved in gross movements with larger force generating capacity. Consequently, the lower limb muscles have more motor units driven by larger alpha motor neurons with higher activation thresholds (Kesar et al. 2018). Furthermore, voluntary contraction increases motor-evoked potential size through increases in cortical and spinal excitability (Di Lazzaro 2004). Therefore, the large relative changes observed in MEP amplitude in the resting FDI muscle after water (10%) and quinine (16%) ingestion (Gam et al. 2015a) will likely have a diminished relative influence in contracting lower limb muscle where MEPs are larger with higher activation thresholds. Indeed, the present data show no effect of quinine ingestion, or water, on MEP amplitude during quadricep contraction which is more relevant to whole-body exercise. Therefore, these data question whether the increase in corticomotor excitability observed in the resting FDI after quinine ingestion (Gam et al. 2015a) is a mechanism that can explain the potential ergogenic effect of quinine ingestion (Gam et al. 2014). Additionally, as tasting a salt solution did not impact quadricep MEPs, the present study does not support the hypothesis that unpleasant tastes can increase corticomotor excitability in a manner that is relevant to exercise performance. However, it should be noted that the present study used single site TMS. While valuable, single site measures only provide insight into corticomotor excitability, and do not reflect other valuable indices of corticomotor reorganization such as changes in representation size or location.

Tasting the bitter compound quinine has previously been shown to improve cycling sprint performance (Gam et al. 2014) and corticomotor excitability (Gam et al. 2015a). Therefore, because quadricep neuromuscular function is a major determinant of cycling sprint performance (Kordi et al. 2017) and changes in corticomotor excitability can lead to changes in muscle function (Collins et al. 2017), we hypothesized that the unpleasant tastes would enhance quadricep neuromuscular function. In contrast, we found no differences in knee extensor neuromuscular function between the unpleasant salt and bitter tastes and the control conditions. Due to the fatigue of completing 13 MVCs within a single session, order effects between the first and second presentation of each condition were detected for some of the neuromuscular function variables. However, for each variable with an order effect, separate analysis of the first and second presentation revealed no significant differences between conditions. Therefore, it appears that the null finding of unpleasant tastes on neuromuscular function is robust despite an order effect in some variables. It is possible that the influence of the unpleasant tastes on neuromuscular function is insufficient until the individual becomes fatigued. Indeed, as taste can only exert an influence on neuromuscular function through a central action, their influence could be amplified in the presence of central fatigue. Supporting this, Khong et al. (2020) showed that mouth rinsing a salt solution, compared to water, immediately before an MVC ameliorated the post-exercise decline in maximum voluntary torque (ES = 0.67) that occurred after 30 min of cycling at 70% of VO_2max_. However, without a direct pre- and post-exercise comparison within a single study, further research is required to determine how fatigue impacts the influence of unpleasant tastes on neuromuscular function.

The present data showed a significantly greater increase in heart rate after mouth rinsing and ingesting the bitter or salty solutions compared to control conditions. However, the data from the no solution and water control conditions show that only 5–6 bpm of the increase in heart rate can be attributed to the unpleasant taste itself. An increase of 14 bpm was caused by moving the arm and tilting back the head, evidenced by the no solution condition increasing heart rate by 14 bpm. A further 4 bpm was attributable to rinsing a solution around the mouth, as evidenced by water increasing heart rate by 18 bpm. Therefore, although the increase in heart rate suggests the unpleasant tastes caused a greater autonomic nervous system disturbance, the small changes observed in heart rate at rest are unlikely to be relevant to exercise performance. Supporting this, mouth rinsing and ingesting quinine has been shown to improve 30 s cycling sprint performance; however, it had no effect on heart rate immediately post-exercise (Gam et al. 2014).

In the present study, participants were able to eat up to 1 h before testing and so were in the fed state when testing commenced. Athletes typically train and race in the fed state and so this ensured ecological validity. However, it is possible that participants’ responses to the unpleasant tastes were diminished, because they were satiated. Indeed, the ergogenic effect of carbohydrate mouth rinsing appears to be dependent on if an individual is pre- or post-prandial. When directly manipulating nutritional state, most research finds carbohydrate mouth rinsing to improve performance to a greater extent when individuals are fasted compared to fed (Ataide-Silva et al. 2016; Fares and Kayser 2011; Lane et al. 2013), although this outcome is not universal (Trommelen et al. 2015). The diminished influence of carbohydrate mouth rinsing in the fed state is likely attributable to reductions in activity of brain regions involved with emotional processing of taste when individuals are satiated (Rolls et al. 2010). However, this response is known as sensory-specific satiety as the decreased neuronal response to satiety is specific to the type of food being eaten, and, therefore, does not occur when a new taste is introduced (Rolls et al. 1986). Consequently, it seems unlikely that recent food consumption in the present study would have dampened the neural response to the unpleasant tastes due to the differences in sensory profile. Nevertheless, it should be noted that studies that have shown an unpleasant quinine solution to be ergogenic, or demonstrate desirable qualities to exercise performance, occurred in the fasted state (Gam et al. 2014; Gam et al. 2015a). However, a clear conclusion about the influence of participants’ prandial state on quinines ergogenic effect cannot be drawn from a handful of studies with divergent methodologies.

The placebo effect is an advantageous outcome of an individuals expected or learned response to a specific stimulus (Beedie et al. 2018), which can directly improve sports performance (Hurst et al. 2020). It is likely that the ergogenic effect of taste, like any ergogenic aid, is attributable to a combination of ‘true’ effect and placebo effect (Best et al. 2021). In research where taste is the independent variable, it is not possible to blind individuals to condition. Consequently, it is difficult to separate the placebo effect from a ‘true’ effect. In the present study, to mitigate the influence of, and measure any, placebo/nocebo effect, we included two control conditions (water and sweet) for the unpleasant tastes. This diversified participants expectations from each condition, preventing easy identification of the researcher’s expected outcome, as is possible with only two conditions. Additionally, we determined participants’ expectations of how each taste would influence neuromuscular function prior to beginning the experiment. This revealed that some participants expected the sweet solution to improve performance (7/20) and the bitter to impair performance (4/20). The nocebo effect evident in the bitter condition highlights that most individuals are unaware of its potential as an ergogenic aid. However, these placebo and nocebo effects were not substantial enough to manifest into changes in neuromuscular function. This could be because most of the participants did not have any prior expectations of how the tastes would influence neuromuscular function (13/20). Consequently, the influence of placebo and nocebo effects on the performance outcomes remain to be established.

## Conclusion

The present data demonstrate that mouth rinsing and ingesting unpleasant salt and bitter tastes caused small changes in heart rate. However, the unpleasant solutions did not influence quadricep neuromuscular function or corticomotor excitability in a rested state. These data question the purported mechanisms via which bitter, and other unpleasant, tastes are proposed to have an influence on exercise performance.

## Data Availability

All data are available from the corresponding author upon reasonable request.

## Abbreviations

ANS: Autonomic nervous system
EMG: Electromyography
FDI: First dorsal interossei
MEP: Motor-evoked potential
MVC: Maximal voluntary contraction
RMS: Root mean square
TMS: Transcranial magnetic stimulation
VL: Vastus lateralis
VM: Vastus medialis
